# Three-Dimensionally Printed Mini Air Scrubbing Cartridges Based on Nano-Graphite for Air Pollution Monitoring

**DOI:** 10.3390/s25010122

**Published:** 2024-12-28

**Authors:** Emiliano Zampetti, Mattia Ammiraglia, Marco Conti, Cassandra Montiroli, Paolo Papa, Daniele Bianconi, Antonella Macagnano

**Affiliations:** Institute of Atmospheric Pollution Research–National Research Council (IIA-CNR), Research Area of Rome 1, Strada Provinciale 35d, Montelibretti, 9-00010 Roma, Italy

**Keywords:** 3D printing, nano-graphite, environmental monitoring, benzene, sensors, nanomaterial

## Abstract

Ecosystems and environments are impacted by atmospheric pollution, which has significant effects on human health and climate. For these reasons, devices for developing portable and low-cost monitoring systems are required to assess human exposure during daily life. In the last decade, the advancements of 3D printing technology have pushed researchers to exploit, in different fields of applications, the advantages offered, such as rapid prototyping and low-cost replication of complex sample treatment devices. In this work, we present the fabrication and testing of 3D printed cartridges based on both commercial photopolymer and a modified version with the intrusion of nano graphite. The air scrubbing performances towards some volatile organic compounds have been investigated, inserting the cartridges into a low-cost monitoring system using a photoionization sensor. In particular, the cartridges were tested in the presence of concentrations of ethanol, benzene, and toluene to evaluate the abatement percentage with and without their use. Although the results have shown that all cartridges abated ethanol and toluene, the abatement of benzene increased 20 times in the case of cartridges based on modified resin with nano graphite. These results could enable their employment to reduce the concentration of interfering compounds in low-cost monitoring systems.

## 1. Introduction

Atmospheric pollution affects important parameters that regulate ecosystems and environments. For these reasons, millions of people worldwide get sick or die from causes correlated to atmospheric pollution, such as stroke, heart disease, pulmonary disease, lung cancer, and respiratory infections [1,2,3]. An important class of air pollutants is volatile organic compounds (VOCs) generated from natural and anthropic sources [4]. Emissions from industrial activities, home cleaning products, cosmetics, and painting products are generally considered the main important VOC sources [5]. In this framework, portable and low-cost monitoring systems are required to assess human exposure during daily life in the workplace, at home, and outdoors with high resolution time. Often, these systems, due to their characteristics, cannot use analytical measurement methods such as offline passive samples or chromatographic analysis [6,7,8]; instead, they increasingly use monitoring systems that exploit low-cost sensors based on nanostructured conductive materials, metal oxides materials, photoionization detectors or electrochemical sensors [9,10,11,12].

However, such systems suffer some issues, for example, the broad-spectrum sensitivity of the low-cost sensor employed could produce measurement error or not usable data [13,14]. To mitigate these problems, many research groups study the possibility of enhancing the system selectivity versus some analytes, removing or reducing the interferent compounds that are not addressed in the case study. Various strategies to handle the sampled air before the measurements imply the use of adsorption, absorption, condensation, and catalytic processes to develop devices that are devoted to this scope [15]. One of the most important devices involves the use of chromatography techniques to separate compounds that are present in a complex mixture of VOCs [16,17]. Another strategy consists of the realization of mini-preconcentrating devices to enhance the sensor response signal to specific compounds [18,19]. Other groups propose the use of innovative analysis algorithms to retrieve usable data from all collected data [20]. In recent years, additive manufacturing (AM), as a well-known 3D printing technology, has allowed us to fabricate objects made of a plethora of materials and shapes designed by CAD software (Freecad 1.0). The use of 3D printing in the industrial research and development sector plays an increasingly important role due to some of its peculiarities, such as the possibility of rapid prototyping, the precision achieved in printing, the availability of printable materials with the possibility of modification or the possibility of designing increasingly complex shapes and structures with smart functions [21,22].

Additive printing techniques generally differ from each other in the method used to deposit or solidify the printable materials on a substrate. Between them, fused deposition modeling (FDM) extrudes by thermal process a filament of thermoplastic materials and deposits it following a specific pathway generated from the CAD file [23]. Stereolithography (SLA) or vat photopolymerization, developed by Charles Hull in 1986, uses an electronically controlled light source (VIS, UV) to photopolymerize a liquid mixture of photo-reactive materials (photo printable resin), using a cross-linking method [24,25,26]. A modified version of SLA is masked stereolithography (MSLA), which uses a digital electronic device to mask part of the light generated from a UV LED matrix to selectively photopolymerize the resin following the CAD file. MSLA low-cost printers use LCD to mask a diffused UV LED light at 409 nm instead of a complicated array of micromirror or electronically controlled laser sources. The precision of these types of printers depends mainly on the LCD resolution. Many research groups proposed 3D printer technology to solve some problems correlated to the cost or the complexity in different research fields. In the field of environmental monitoring, 3D-printed devices were developed to sample, treat, and remove pollutants in water depuration applications or in air quality monitoring systems [27,28]. In the fields of biology and chemistry, to develop reaction cells or microfluidics channels, mix separate compounds to optimize the costs and the occupied volume [29]. Moreover, in mechanical or materials engineering, 3D printing technology is used to fabricate devices or structures based on modified photopolymer, reinforced organic or inorganic materials [30,31]. Three-dimensional technology can be used to remove benzene from the air using a developed electro-absorber reactor [32,33].

In this work, we present 3D MSLA printed cartridges based on the commercial water-washed resin (WW) and modified resin tested in the presence of VOCs. In particular, the cartridges were tested in the presence of concentrations of ethanol, benzene, and toluene. The measurements were conducted by placing the different cartridges upstream of a PID sensor with a broad sensitivity spectrum. One of the most important goals of this study is to assess and measure the potential reduction (or abatement) in the concentration of the targeted analyte reaching the sensor after its interaction with the printed cartridges. The cartridges were produced using a modified version of a commercial resin (the details are outlined in the Section 2) by incorporating nano graphite particles. This graphite inclusion was specifically designed to enhance the cartridge’s adsorption and retention capabilities for the selected VOCs. Graphite, a carbon material well-known for its adeptness in adsorbing organic volatile compounds (VOCs) [34], was selected for this study due to several factors related to the operational conditions of the produced cartridges and a cost–benefit analysis [35,36]. On the other hand, graphene, a two-dimensional structure of carbon atoms forming a single layer, is well known for its exceptional strength and conductivity. In contrast, graphite occurs naturally as a carbon material consisting of multiple layers of graphene stacked on top of each other. Despite this layered arrangement, graphite lacks enhanced conductivity and strength characteristic of graphene. For the specific objectives of this research, the superior mechanical properties of graphene over graphite are unnecessary [37]. The decision to opt for graphite instead of graphene was influenced by a variety of factors beyond just mechanical properties. Primarily, the cost was a significant factor; the production of graphene from graphite entails several expensive techniques, making graphene considerably more costly [38,39]. In terms of surface interaction with the incoming analyte flow in the modified cartridges, it is well-documented that graphene offers a substantially larger active surface area compared to graphite, approximately ~2600 m^2^/g versus the 1 m^2^/g of graphite [40].

To address the disparity in active surface area, graphite nanoparticles were utilized to maximize their active surface area [41,42]. Furthermore, graphene possesses chemically reactive edges and surfaces that can react with the incoming analyte flow, potentially altering the structure of graphene and compromising its adsorption efficiency over time, whereas graphite exhibits relatively high resistance to chemical reactions and generally behaves inertly. Therefore, the decision to employ graphite nanoparticles over graphene was made after careful consideration of the system requirements discussed so far.

Moreover, graphite exhibits exceptional thermal stability, is capable of withstanding temperatures exceeding 3600 °C, and is commonly used as a reinforcing element in various materials [43]. The incorporation of graphite enables the restoration of the initial adsorption characteristics of printed cartridges by allowing exposure to elevated temperatures beyond those typical of cartridges produced using conventional WW commercial resin formulations (in our case, <100 °C). Experimental findings indicate that cartridges containing graphite in every formulation produced can endure temperatures up to 250 °C for 3 h without structural compromise, in stark contrast to WW cartridges, which deform under temperatures exceeding 80 °C, losing mechanical integrity. Subsequent investigations reveal that these elevated temperature conditions facilitate the desorption of the entrapped analytes, restoring the cartridge for multiple experimental iterations.

The main findings outlined in this study related to the development of VOC retention systems referred to as cartridges, obtained by optimization of 3D printing parameters and materials. These cartridges were obtained using a commercial resin formulation known as Water Washable (WW), which was demonstrated to have a reduced environmental impact by being easily removed using water as a solvent before curing. Thus, this formulation allows for the direct rinsing of the produced parts in distilled water, eliminating the need for solvents with higher environmental impacts, such as ethanol. The 3D-printing parameters were optimized in order to obtain cartridges with channels as small as 1 mm while maintaining the structural integrity of the structure as the number of layers being printed increases. Subsequent optimization of printing procedures enabled the fabrication of the same structures using the WW resin filled with nano graphite particles, enhancing the retention properties of the cartridges for the VOCs being analyzed. Experimental results demonstrate how the graphite functionalization of the obtained cartridges allows for improved interaction with the analyzed compounds, specifically ethanol, toluene, and benzene. Furthermore, the experimental data highlights the retention characteristics of the cartridges and the nano graphite within the modified cartridges. These observations have led to the proposal of an adsorption mechanism within the obtained cartridges in alignment with the collected data. Further investigations into the retention mechanisms and properties of the obtained modified cartridges are necessary for a better understanding of the established system and to pave the way for the potential development of composite resins through LCD-3D printing for environmental monitoring application use.

## 2. Methods and Materials

### 2.1. Cartridge Design

**Figure 1:**
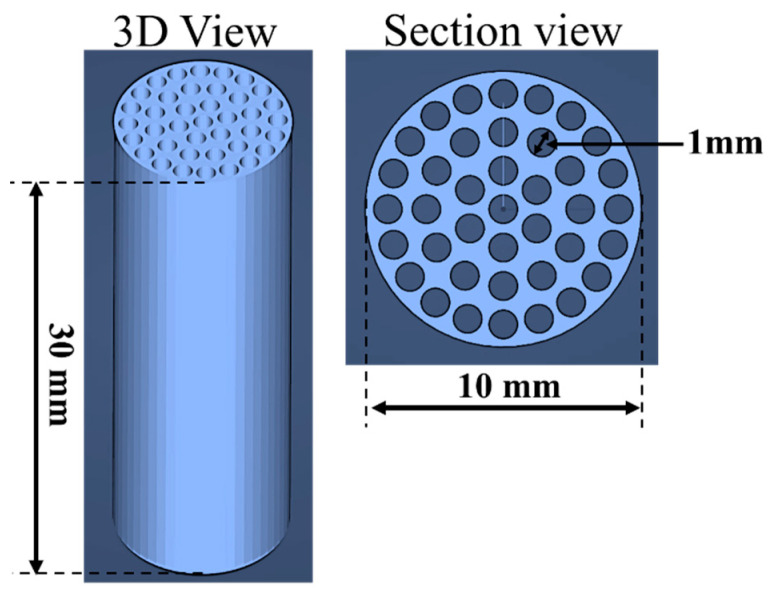
Cartridge 3D CAD image. The cartridge was 30 mm in height, with a diameter of 10 mm, and it had 39 cylindrical channels 1 mm in diameter.

As mentioned in the introduction, our intent was to study the possibility of using 3D printing technology to develop and test a mini cartridge working as a scrubber (or abatement) device useful in low-cost air pollution monitoring systems. In order to facilitate the utilization of the cartridge within a monitoring system, the shape and dimensions were predetermined during the design phase to adhere to several crucial technical specifications, such as: (a) the overall dimensions should be in the order of few centimeters to be inserted in a portable, low-cost sensor system, (b) it should be simply connected by a commercial tubing adaptor, (c) the shape and structure should guarantee good mechanical performance during all tests carried out under a flux generated by a low-cost pump (e.g., mini membranes pump) with neglectable pressure drops. For these reasons, we opted for a cylindrical shaping cartridge with circular channels to facilitate the flow passage. In Figure 1, the 3D CAD image of the designed cartridge is reported, showcasing a height of 30 mm, a diameter of 10 mm, and 39 cylindrical channels with a diameter of 1 mm.

The designed cartridge model has a total inner cartridge surface area of about 36 cm^2^ (calculated as 39 times the lateral surface area of single cylindric channel) that results in about two times greater than maximum lateral area of a simple cartridge having a single channel of 10 mm of diameter. The last design phase was the cartridge 3D file conversion by a free LCD slicer software (CHITUBOX Basic V2) to be ready for the printer.

### 2.2. Preparation of Composites Photopolymer

This study employed a commercially available water-washable transparent resin (WW) developed by MulticompPRO (cod. MP004402). To enhance the physio/chemical interaction capability of the cartridges towards the volatile organic compounds under investigation, graphite nanoparticles, with an average particle size of 500 nm, purchased from Sigma-Aldrich-Merck (cod. MKBB1704) were mixed into the resin at varying concentrations. The obtained composite materials, WW + G, have been printed using the same process and parameters as those for standard WW. Specifications of the sample cartridges have been reported in Table 1; the nano graphite concentrations are expressed with the weight ratio percentage defined as follows:

(1) $$C_{G/WW}=\left(\frac{m_{G}}{m_{WW}}\right)\times 100\;[\%]$$

where $m_{G}$ and $m_{WW}$ are the weighted mass of the graphite nanoparticles and the water washable resin, respectively. $C_{G/WW}$ is the percentage concentration of the nano-charge included in the standard formulation of the resin used. Table 1 presents the measured weights for the graphite nanoparticles and WW resin across various tested concentrations; the data refer to about 60 g of total resin inserted in the 3D printer container. The adopted amount of total resin depends on the minimum volume that is required for the printer configuration to ensure the homogeneity of printer plate covering.

In Table 1, the data error values were calculated, considering the method and the resolution of the analytical balance (TE2145 by Sartorious) used during all sample preparation.

The resin volume remains consistent across all produced cartridges, aligning with the minimum volume specified by the printing parameters employed. Through a series of experimental observations, the upper threshold for nano graphite concentration in the cartridges was determined. Notably, a concentration of 0.2% emerged as the maximal level to uphold the mechanical integrity of the final structure within the specified printing parameters of this study.

**Figure 2:**
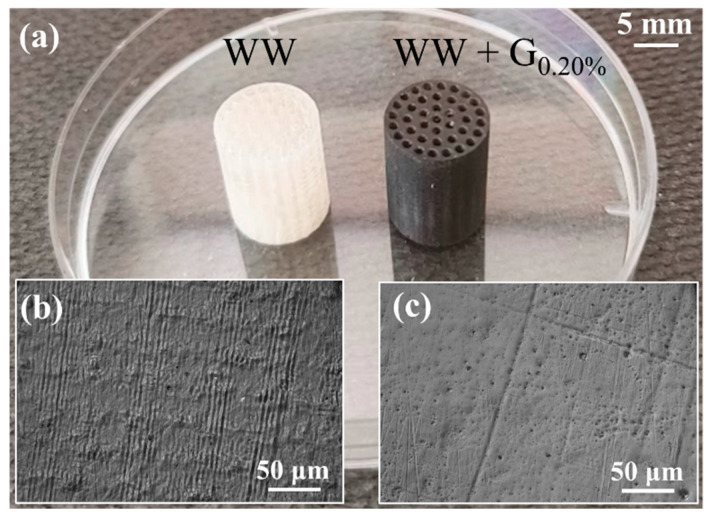
(**a**) Photograph of the WW and WW + G_0.20%_ 3D printed cartridges. (**b**) Microphotographs of the surface of the WW cartridge and (**c**) WW + G_0.20%_.

The procedure for preparing the graphite-modified resins entails the direct addition of graphite particles into the WW resin in small quantities under magnetic stirring conditions. The nano graphite inclusion level in the WW + G cartridges has been fixed to assess the influence of graphite incorporation on the interaction of the three analytes—ethanol, benzene, and toluene—while safeguarding the mechanical properties and structural integrity of the printed cartridges in terms of their three-dimensional growth during the printing process. As mentioned in the Section 1, to develop the cartridges, we have used a low-cost LCD 3D printer (Mars 3 Pro by ELEGOO) that uses an 8 inches 4K LCD (4098 pixels × 2560 pixels) with an estimated resolution of about 35 µm, a minimum layer thickness of 25 µm and 409 nm of UV source. The parameters used during the printing process were the same for all cartridges, we used an exposition time of 10 s, obtaining a total printing time of about 3 h for each type of cartridge. At the end of the printing, the cartridges were washed in distilled water bath under ultrasonic waves, and then a curing process under UV source (409 nm) lasting 15 min was used to complete the sample fabrication. To obtain more representative experimental test results, we have developed and tested six replications of each cartridge typology. An example of the developed WW and the WW + G cartridges is shown in Figure 2a.

**Figure 3:**
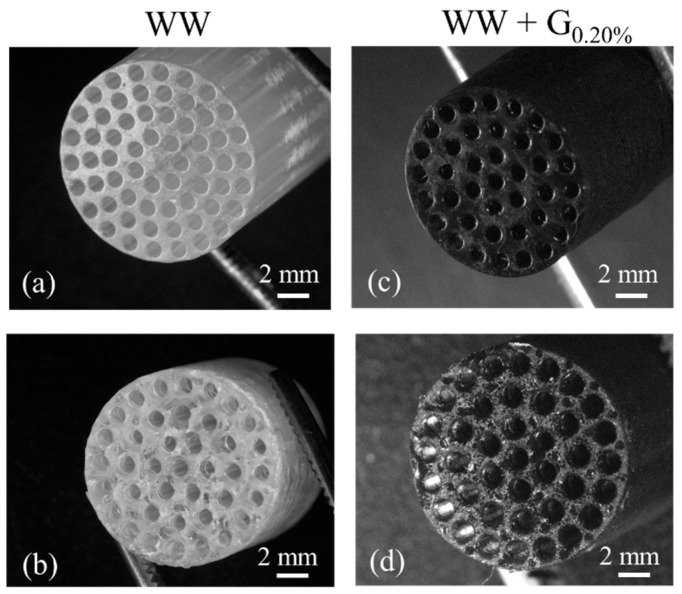
Microphotographs of cartridge 3D structures. Details of the WW and WW + G_0.20%_ channels (**a**,**c**). Details of the WW and WW + G_0.20%_ channels at the cutting surface (**b**,**d**).

The average weight of the developed cartridges (WW and WW + G) was 1.65 g ± 0.1 g, with an average diameter of 10.0 mm ± 0.1 mm and an average height of 30.0 ± 0.1 mm. The WW cartridges appeared transparent to visible light due to the formulation of the standard commercial resin. Instead, the cartridges modified with graphite inclusion exhibit a black appearance. The addition of graphite, even at the minimum amount, has significantly altered how the composite material interacts with visible light [43]. In Figure 2b,c are reported digital microphotographs acquired by DM2700M (by Leica) that denote superficial differences between the WW and WW + G cartridges. On the surface of WW cartridge (Figure 2b), it is possible to see a geometric pattern that could be due to the LCD printer resolution. In fact, the pitch of the pattern is very close to the dimension of the LCD pixel. Conversely, the WW + G surface appears smoother, and the patterning is not evident. These phenomena could be associated with an optical effect due to the presence of graphite that acts as a diffraction material during the resin photopolymerization. A microscope visual inspection performed by 3I INVESTA (by Leica) highlighted that the 3D structure of the different cartridges seems to be the same, and even the integrity of the channels is the same between the WW and the WW + G formulation (see Figure 3).

Figure 3a,c show the microphotographs of the cartridge WW and WW + G_0.20%_ with a detail of the top cartridge surface (the last printed layer). Figure 3b,d show the internal structure microphotograph obtained by cutting the cartridge in the middle. The surface of the WW + G cartridges appears marginally more porous; this effect could be due to the presence of solid external particles within the resin. In addition, during the cutting of WW we noticed a production of small pieces of cartridge, on the contrary, for WW + G, the cutting was “clearer” without any observable sample destruction. To study the microscopic aggregation and distribution of the nanoparticles of graphite inner 3D printed materials, different thin (0.5 mm) layers of samples-based WW and WW + G resin have been printed and analyzed.

**Figure 4:**
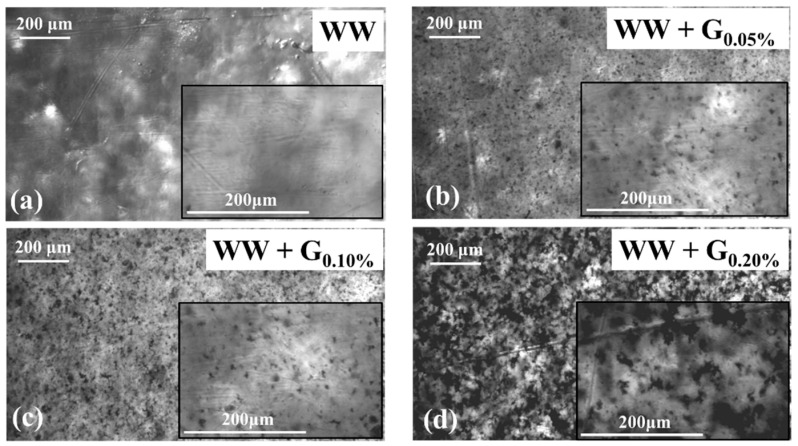
Microphotographs captured in transmission mode of WW and WW + G samples of 3D-printed thin films (about 0.5 mm thick). Magnified pictures highlighted the graphite aggregation.

Upon magnification using an optical microscope in transmission mode functioning (FE2540 model by EUROMEX), the incorporation of graphite nanoparticles becomes evident, as depicted in Figure 4. The escalation in particle inclusion in the resin corresponds to an increase in graphite concentration, showing a homogeneous distribution across all WW + G cartridges. Notably, a higher particle inclusion leads to intensified graphite agglomeration within the polymerized resin, evidenced by a greater number of aggregation points in the 0.20% loaded cartridges compared to the 0.05% ones. However, it is important to note that the distribution of graphite remains consistent across the surface of all cartridges, even in the case of WW + G_0.20%_

For a more comprehensive exploration of the surface effects resulting from various concentrations of graphite in 3D-printed cartridges, contact angle wettability measurements were conducted across the thin layer samples previously mentioned, using a SURFTENS-universal contact angle measuring apparatus (obtained from Exacta + Optech Labcenter S.p.A., Italy) [44].

### 2.3. Measurement Setup and Analytes

**Figure 5:**
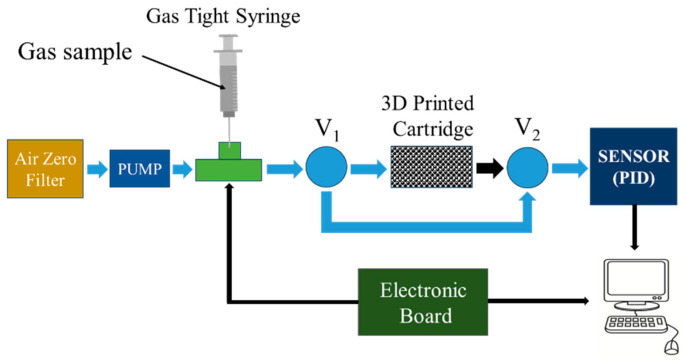
Measurement set-up scheme. The syringe surges a fixed gas volume (few µL) from the headspace of a vial containing the liquid phase of analyte.

In order to evaluate the interactions and scrubbing performances of the 3D-printed cartridges with targeting gas molecules, a suitable measurement system has been developed. As depicted in the scheme of Figure 5, it consisted of the following main parts: (a) Gas tight syringe (25 µL syringe by Hamilton), (b) mini DC pump connected to an air zero filter, generating the main air flux (fixed to a 300 mL/min), (c) “T-shape connector” with a sealed needle orifice for gas injection, (d) a sealed 3D printed cartridge accommodation, (e) PID sensor (PID-AH by Alphasense) accommodated into a suitable chamber, (f) two-way electro valves (V_1_ and V_2_) and (g) an electronic board, that controls the syringe surging and injecting volume, acquire the sensor signal and transmit the data to PC unit. In particular, we have paid more attention to the 3D cartridge accommodation that was made using a Teflon tube with the inner diameter equal to the cartridge diameter and to avoid any kind of flow losses, a polyimide sealing resin (23817 suitable for gas chromatography, by Supelco) was used to seal all the interstices between the cartridge and the inner tube surfaces (on the front and the bottom of the cartridge). A fixed gas volume V_sample_ (few microliters) was surged by the syringe from the headspace of a vial containing the liquid phase of the analytes under test. When V_sample_ is injected into the air flux, with a fixed injection time, the resulting concentration peak revealed from the PID could flow through the cartridge or bypass it (depending on the v1 and v2 status. In such manner, by changing the injected volume and/or the injection time (from few seconds to minutes), it was possible to change the peak properties such as peak height and peak width. We have adopted this strategy to carry out experiments to avoid the sensor response saturation (in our case, hundreds of ppm), long recovery times for each test session, and sensor aging [45] For all tests performed, we have used three target gases: ethanol, benzene, and toluene purchased by Sigma-Aldrich-Merck (Cod. 1070172511, Cod. 244511, Cod. 319953, respectively). As previously mentioned, our goal was to study the abatement effects of the 3D-printed cartridges. The data for the output of the PID sensor were reported in arbitrary units (au) for simplicity. Moreover, it is possible to convert the sensor response in voltage or in concentration by several experiments devoted to the calibration, but it is not necessary in our case because we evaluate the cartridge performances by a percentage of signal variation with and without the cartridge insertion.

**Figure 6:**
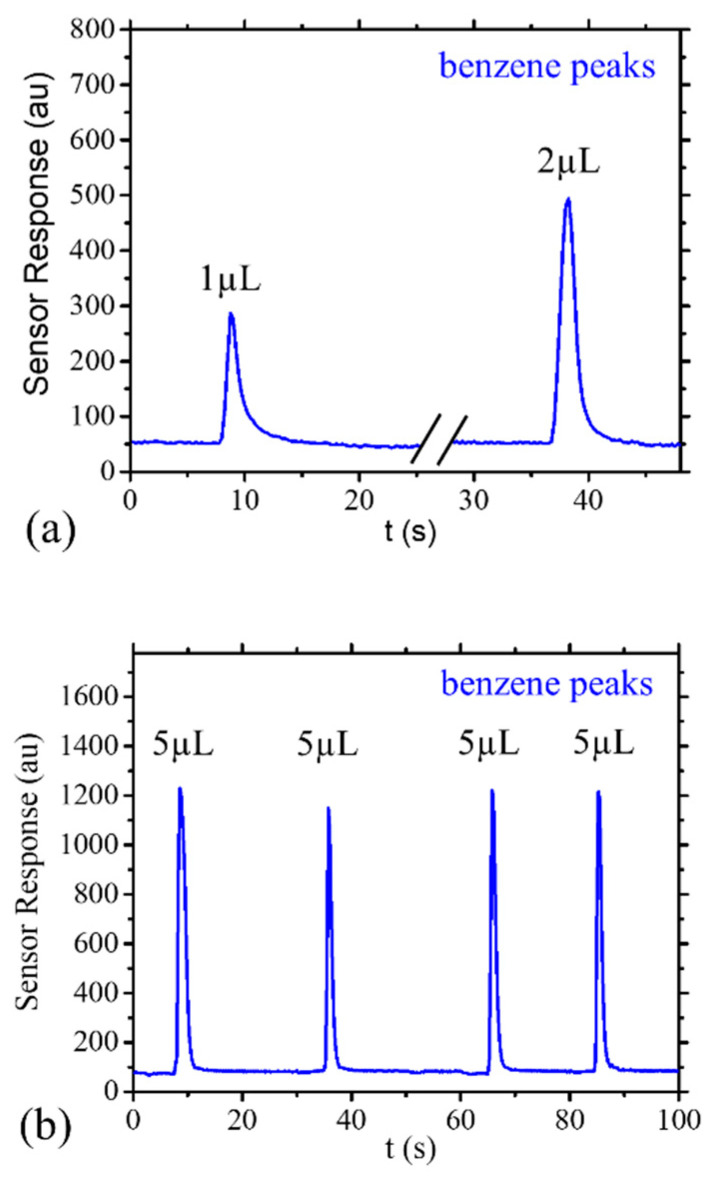
Example of sensor response to the generated peaks after the dilution performed by the measurement setup. (**a**) Sensor response for 1 µL, 2 µL of injected benzene. (**b**) Sensor response for four consecutive 5 µL of injected benzene.

The sensor response to the air, without any injection, at 25 °C ± 0.5 °C (controlled temperature of the box-contained measurement set-up) was in the range of 70 to 85 (au) this value depends on both performances of the zero-air filter and the specifications of PID-AH sensor (e.g., temperature, humidity, power voltage, sensor aging …). As is well known, PID sensors have a rapid response (<1 s) and a broad spectrum of sensitivity and, for this reason, are generally used as sensors for total VOC measurements. In first approximation, its response is the sum of the single response to each compound that composes the air mixture under measure. To obtain a stable value of analyte vapor pressure in the vial headspace, during the experiments, the vials were maintained at constant temperature of 24 °C ± 0.2 °C using a thermal controlled bath. Starting from the NIST phase change data [46], it is possible to calculate the concentration of saturated vapor pressure for the three analytes. The resulting concentrations were about 73 × 10^3^ ppm for ethanol, about 120 × 10^3^ ppm for benzene, and about 36 × 10^3^ ppm for toluene. The information on the saturated vapor pressures of all analytes was utilized to normalize the signal results for assessing the percentage of result abatement. In Figure 6a are reported examples of sensor response to 1 µL and 2 µL of benzene, injected with an infusion time of 4 s for each one. Figure 6b reports four consecutive peaks of benzene measured when 5 µL of benzene was injected with an infusion time of 4 s each. All the peaks have a mean width value of 4 s and a mean peak height of 1085 (au), with an estimated error of about ±10% for the peak area. For all the experiments, we have fixed the infusion time at 4 s. Figure 6 demonstrates that the measurement setup reduces (by a dilution) the concentration peak about 10^−4^ times, avoiding the sensor signal saturation.

In order to evaluate the abatement performance of each cartridge towards the selected VOCs, an abatement percentage (Equation (2)) has been defined and analyzed.

(2) $$\mathrm{Abatement}\;(\%)=\left[\frac{A_{X}-A_{Y}}{A_{X}}\right]\times 100$$

where *A_X_* is the sensor response peak area value reading after an analyte injection without cartridge, and *A_Y_* is the sensor response peak area value reading after an analyte injection with cartridges (WW or WW + G) insertion. The peas area was calculated by using an open-source signal processing tool (Scilab, https://www.scilab.org/about/company, accessed on 1 January 2024). In addition, the area abatement average data were normalized to the toluene concentration (the lower one, due to the different saturated vapor pressure) and to sensor coefficient response [45].

## 3. Results and Discussions

**Figure 7:**
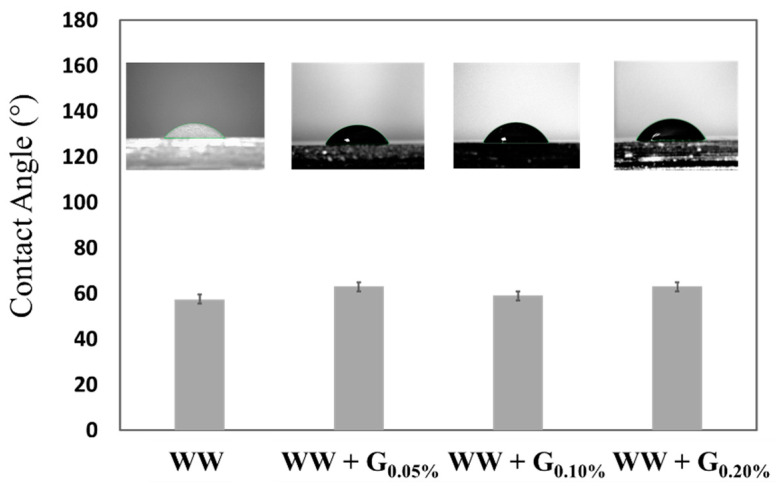
Contact angle analysis conducted on WW and WW + G-based 3D printed thin layer. By leveraging a software function integrated into the instruments, a drop volume reproducibility of 0.1 µL was attained for every analyzed sample.

Contact angle measurement results indicate a notable hydrophilicity of the material surface for all samples, with consistent contact angles of approximately 60° observed across multiple measurements, see Figure 7. In a recent investigation by Wang et al., a contact angle of 98.3° was measured on graphene monolayers and a few-layer graphene stacks synthesized via chemical exfoliation of natural graphite flakes [47].

The uniformity of contact angle values obtained in our samples suggests, in agreement with the results presented by Wang et al., that the cartridge surfaces lack exposed graphite agglomerates but instead demonstrate effective dispersion and integration of graphite within the polymerized lattice. Consequently, as the graphite particles are homogeneously distributed within the resin matrix, contact angle measurements exhibit no discernible variations across different printed materials, regardless of graphite inclusion.

One of the purposes of this work is to determine whether the filter cartridges produced using the standard commercial WW resin and those modified by the introduction of graphite nanoparticles at various concentrations within the resin pre-polymerization (WW + G) exhibit capable characteristics of retaining the analyzed molecules inside the cartridge. Subsequently, an evaluation of the percentage of reduction of the corresponding sensor signal was considered, as previously described. Therefore, the cartridges were designed to function as filters, capable of retaining the compounds under analysis through weak interactions with the internal surface of the cartridge [33,35]. The analytes selected for this study were benzene, toluene, and ethanol. To accomplish this objective, the fabricated cartridges (WW and WW + G) were configured with a cylindrical external architecture featuring longitudinally aligned channels, as previously described. These apertures exhibit a diameter of 1 mm, consistent across both the conventional WW resin cartridges and those enhanced by the integration of graphite nanopowders at differing concentrations investigated. The internal channels facilitate a steady passage of introduced target analyte, increasing the effective surface area of the internal framework [48]. The reduction degree of the compounds was evaluated by comparing the flux passing through the printed cartridges before reaching the sensor with the one bypassing the cartridge.

**Figure 8:**
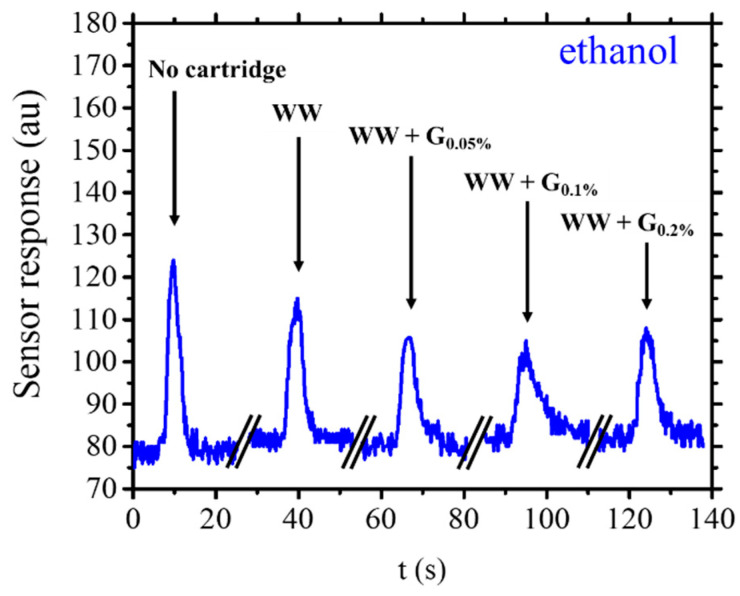
Example of sensor response for 5 µL of injected ethanol (for an infusion time of 4 s) without and with cartridges WW and WW + G types.

Upon the introduction of ethanol into the sensor/WW cartridge system, a decrease in signal intensity of around 26% is detected compared to the sensor alone, as delineated by Equation (2). The WW cartridges, shaped with a specific channel design for this investigation, exhibit a capacity for partial ethanol molecule adsorption. Signal attenuation is also observed when employing the WW + G cartridges within the system, manifesting as reductions of around 25% for all three different graphite concentrations investigated. The comparable nature of these values suggests a consistent trend. It is plausible to hypothesize that minimal variability exists in the efficacy of WW + G cartridges in ethanol adsorption across diverse nano graphite concentrations within the experimentally observed spectrum. The data suggests that ethanol absorption by both the standard WW and graphite-modified cartridges exhibits similar behavior across the entire range of concentrations investigated. Thus, it can be asserted that the interaction of ethanol molecules within the two cartridge types is uniform, indicating that ethanol lacks significantly distinct interactions with the graphite present in the modified cartridges. Figure 8 shows the difference between the cartridges when ethanol is used in the system. The calculated area under the peaks for ethanol in the different cartridges is comparable among them; the main difference lies in the various elution times, and peak broadening for the signals could be attributed to the cartridges loaded with graphite. This could suggest an extremely weak nature of ethanol interaction with the graphite, which does not alter the recorded signal abatement values. Therefore, the only noteworthy interactions appear to be those that ethanol has with the residues of the polymerization of the WW resin itself.

**Figure 9:**
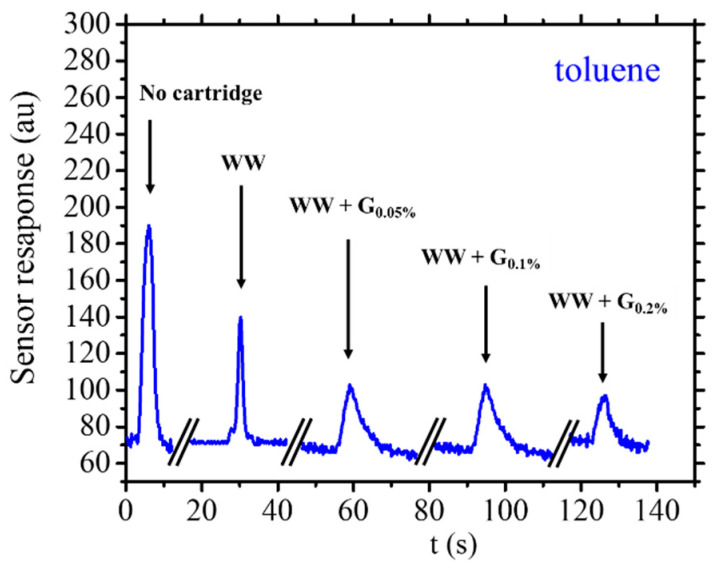
Example of sensor response for 5 µL of injected toluene (for an infusion time of 4 s) without and with cartridges WW and WW + G types.

Regarding the sensor response to the introduction of toluene into the system, a value of 35% reduction is observed when utilizing the WW cartridge. This reduction aligns closely with the reduction exhibited in response to the ethanol molecule. Consequently, the data suggests that the WW cartridge demonstrates comparable efficiency in retaining both ethanol and toluene molecules. Notably, when employing WW + G cartridges, a substantial divergence is witnessed in the behavior between toluene and ethanol. A substantial signal reduction is observed across the entire concentration range of graphite used in the WW + G cartridges. Specifically, an abatement of approximately 55% was calculated for the WW + G_0.05%_ and WW + G_0.10%_ cartridges and 71% for the WW + G_0.20%_ cartridges using equation 2. These findings suggest that toluene adsorption increases with the concentration of graphite in the cartridges. Therefore, besides interacting with the polymeric nature of the cartridge matrix itself, toluene can additionally be retained if graphite particles are present within the resin. Consequently, there exists a dual nature of retention in the case of toluene, a contribution from the resin of the cartridge matrix and a contribution attributed to the presence of the graphite second phase dispersed within the polymeric phase, Figure 9. In the case of toluene, similar to what was observed with ethanol, there is a broadening of the peaks following the introduction of graphite into the cartridge. While it is reasonable to hypothesize a similar behavior with toluene, there is an additional decrease in the signal, indicating an increase in abatement due to the graphite nanoparticles dispersed in the polymeric matrix of the cartridges.

**Figure 10:**
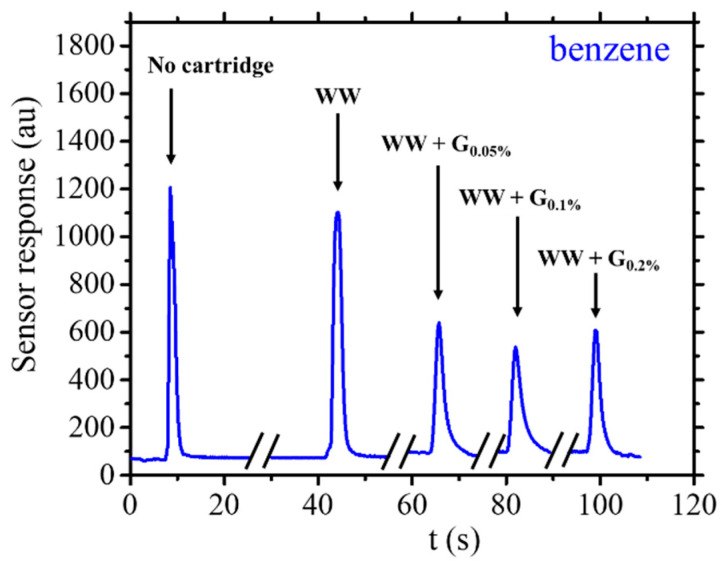
Example of sensor response for 5 µL of injected benzene (with an injection time of 4 s) with and without cartridges WW and WW + G types.

In Figure 10, the sensor response to benzene with and without cartridge is reported. Reports indicate that signals attributed to benzene exhibit higher counts compared to toluene and ethanol. The signal intensity is intricately linked to the sensor’s response to the specific molecules, which is notably stronger for benzene than for the other two molecules. Furthermore, all three analytes are employed at the same rate. It is observed that under identical external pressure, temperature, and humidity conditions, the saturated vapor pressure of benzene surpasses that of toluene and ethanol. Consequently, the sensor signals were normalized using correction factors that account for the aforementioned factors. In the scenario where benzene passes through the WW cartridge to reach the sensor, no substantial signal reductions are observed compared to the system without using any cartridge. The signal reduction recorded is around 5%, which is practically negligible, especially considering the instrumental error. When the WW + G_0.05%_ cartridge is utilized, a significant signal decrease of around 75% is noted at the detector, also calculated by Equation (2). A further decrease is observed with the WW + G_0.01%_ cartridge (doubling the graphite concentration in the cartridge), amounting to 85%. A similar value of 75% is reached by the WW + G_0.20%_ cartridge. Hence, the experimental data suggest that the benzene molecule is not effectively treated by the WW cartridge but is substantially retained by the WW + G cartridges, with a slight increase from the 0.05% graphite concentration in the cartridge to the 0.1% concentration, which reaches similar value as the cartridge loaded with 0.2% in graphite. From the observed concentration range, it can be deduced that benzene molecules exhibit some interactions with the WW + G cartridge but not with the WW cartridge, unlike toluene and ethanol molecules, for which there was still a reduction (even though smaller compared to the graphite-loaded cartridges).

**Figure 11:**
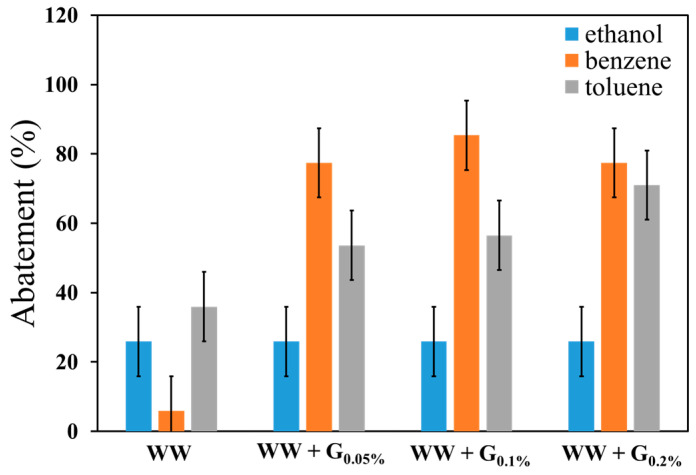
The abatement percentage of the different tested cartridges WW and WW + G types.

A comparative analysis of ethanol, benzene, and toluene is presented in Figure 11 to elucidate their distinct behavioral characteristics.

The experimental results demonstrate a decrease in the signal of the 3-analyte upon the introduction of the WW printed cartridge preceding the sensor. Specifically, a notable reduction is observed when ethanol and toluene are introduced into the system. Concerning benzene, the signal reduction calculated when the WW cartridge is used is minimal and essentially negligible. Overall, for the three compounds examined, a reduction increase is noted between the WW and WW + G cartridges (across the entire range of experimentally tested concentrations). In the case of benzene, a reduction enhancement of 75 ± 10% to 85 ± 10% is observed, 35 ± 10% to 65 ± 10% for toluene, and 25 ± 10% for ethanol when using a modified graphite cartridge rather than a standard WW cartridge, always referring to the concentration detected by the sensor without the use of any cartridge (see Equation (2)). It is important to note that the difference in signal intensity is mainly due to the sensor’s interaction with the different molecules, as well as a correction factor applied for the calculation of the analyte’s ppm concentration based on vapor pressure and the volume introduced into the cartridge + sensor system. Furthermore, no substantial differences are observed when comparing cartridges loaded with graphite at different concentrations. Analyzing the reduction relative to a direct comparison between WW cartridges and those loaded with graphite at 0.20%, 0.10%, and 0.05%, as shown in Figure 11, can provide additional insights.

Overall, it was noted that the incorporation of nano graphite powder in modified cartridges led to an enhanced abatement percentage in sensor signals across the three analytes studied—ethanol, benzene, and toluene. Furthermore, the use of WW cartridges containing the standard resin formulation demonstrated the capture of ethanol and toluene molecules with a signal reduction of approximately 30% each. In contrast, distinct behavior was observed for benzene molecules, which exhibited a signal reduction solely when WW + G. cartridges were utilized in conjunction with the detection sensor. This observation provides insight into a possible detention mechanism of the developed cartridges [49]. However, this is considered a preliminary hypothesis, and further research is required to enhance the comprehension of the interaction between the cartridges (the standard one and the one with graphite inclusions) and the molecules introduced into the system, including ethanol, toluene, and benzene. The decrease in signal detected by the sensor when the printed cartridge is positioned ahead of it implies the presence of interactions between the cartridge and the molecules within the substance passing through the cartridge.

Primary insights derived from the experimental findings reveal different behaviors between benzene and toluene when the WW cartridge, lacking graphite functionalization, is utilized within the system. Notably, toluene demonstrates retention by the cartridge, contrasting with benzene; the signal reduction of toluene when the WW cartridge is used could be ascribed to steric encumbrance or chemical/physical interactions [50]. Despite both molecules bearing planar aromatic rings and sharing structural similarities, benzene shows no retention in the WW cartridge. The experimental results concerning the reduction of toluene signals suggest that the prevailing interaction mechanism may be chemical/physical in nature, given the comparable steric dimensions of the two molecules [36]. Further experimental evidence is provided by the data concerning the ethanol molecule, which exhibits behavior like toluene and is once again markedly different from benzene when the WW cartridge is employed. Ethanol, significantly smaller in size compared to toluene and benzene, suggests that its retention solely within the WW cartridge may be attributed to a chemical/physical interaction rather than steric encumbrance [51]. The WW resin used for the 3D printing of each cartridge is an acrylic resin with an inclusion of epoxy-based oligomers, in which residues of the acrylic groups post-polymerization exhibit carboxylic polar groups, indicating that the hypothesized chemical/physical interaction based on experimental evidence is likely polar in nature [52]. Ethanol’s molecular structure includes an -OH group, imparting polarity, and a hydrocarbon -CH_3_ CH_2_ portion, rendering it nonpolar. In contrast, benzene is a nonpolar molecule with an isotropic charge distribution in the aromatic ring, making it nonpolar. Toluene shares the same aromatic ring as benzene, but the presence of the methyl group slightly alters its electronic structure [53]. In toluene, the bond between the ring and CH_3_ group is slightly polarized towards the ring due to the small difference in electronegativity between the sp^2^ carbon of the ring and the sp^3^ carbon of CH_3_ [54].

The potential interpretation of the experimental data concerning the use of the WW cartridge in the cartridge/sensor system suggests an electrostatic interaction of a dipole–dipole or induced dipole–dipole nature [41]. In this scenario, ethanol and toluene (with the partial distortion of the electric field favoring hyperconjugation of the methyl group) manage to establish weak bonds with the carbonyl residues of the polymerized resin and be retained as they pass through it. Benzene, being an isotropic molecule, is unable to participate in such interactions, leading to no decrease in the signal reaching the sensor when the WW cartridge is employed. In contrast, when the cartridge loaded with WW + G nanoparticles is used, a reduction of around 75–85 ± 10% of the signal for the benzene molecule is observed. Additionally, a signal reduction is also noted for ethanol and toluene molecules, greater than that observed when using the standard WW cartridge. The interaction mechanism of the cartridge with benzene, in this case, can be attributed to the presence of graphite nanoparticles incorporated into the resin matrix and present at the resin interface [55]. The literature extensively discusses the ability of benzene to interact with the stacked planes of graphite, interacting through the aromatic ring current with the graphene plane [49]. Therefore, benzene interacts planarly with the graphene planes within the graphite, leading to an experimental signal decrease when WW + G cartridges are used. The same behavior is observable for toluene with cartridges modified with graphite (the mentioned abatement is around 55–70 ± 10%). This behavior can be attributed to the dual nature of interactions of toluene with the cartridge: one of a dipole–dipole nature with the carbonyl residues of the resin and one attributable to the aromatic ring interaction with the graphite planes [56]. For ethanol as well, an increased signal reduction is observed when transitioning from WW cartridges to WW + G cartridges, although more modestly compared to benzene and toluene reductions. Further investigation and analysis may be needed to fully explain this observation.

## 4. Conclusions

Three-dimensional MSLA printing technology has been employed to develop air-scrubbing cartridges. A 3D cartridge model was designed and then printed using both commercial resin (WW) and nano graphite modified resin (WW + G) with three different levels of graphite intrusion (0.05%, 0.10%, and 0.20% in weight percentage). To study the WW and WW + G material scrubbing performances, each cartridge was inserted in a measurement setup based on a low-cost PID sensor and tested in the presence of concentrations of ethanol, benzene, and toluene. To calculate and compare the analytes scrubbing performances of the single cartridge, the abatement percentage is defined as the relative sensor response variation with and without cartridge use (the lower the signal measured with the cartridge, the higher the percentage of reduction). For both ethanol and toluene, all the cartridges showed an abatement of around 30 ± 10%. A higher value of 55–70 ± 10% was reached for toluene when the WW + G cartridge was used. The standard WW cartridges are not capable of reducing the concentration of benzene, but the WW + G cartridges have this capacity and can achieve a value of about 75–85 ± 10%. The results highlighted that WW + G cartridges exhibit a significantly higher adsorption capacity compared to WW cartridges, especially for toluene and benzene.

Three-dimensional MSLA WW + G cartridges emerge as a promising technology for the realization of low-cost monitoring systems employed as scrubbing devices for VOCs, in particular for benzene and toluene. The ability to fabricate complex structures made of different functionalized materials using 3D MSLA technology opens new prospects for the development of high-performance composite materials with potential applications in several sectors.

## Figures and Tables

**Table 1:** Composites photopolymer for 3D cartridges. Where $m_{G}$ and $m_{WW}$ are the weighted mass of the graphite nanoparticles and the water-washable resin, respectively. $C_{G/WW}$ is the percentage concentration of the nano-charge included in the standard formulation of the resin used.

Sample Cartridge Label	m_G_ [mg]	m_WW_ [mg]	C_G/WW_ [w/w%]
WW	0	60 × 10^3^ ± 0.1	0
WW + G_0.05%_	30.0 ± 0.1	60 × 10^3^ ± 0.1	0.05 ± 0.17 × 10^−3^
WW + G_0.10%_	60.0 ± 0.1	60 × 10^3^ ± 0.1	0.10 ± 0.17 × 10^−3^
WW + G_0.20%_	120.0 ± 0.1	60 × 10^3^ ± 0.1	0.20 ± 0.16 × 10^−3^

## Data Availability

All data that support the findings of this study are available after the reasonable request to the corresponding author.
